# A study of fluid dynamics and human physiology factors driving droplet dispersion from a human sneeze

**DOI:** 10.1063/5.0032006

**Published:** 2020-11-01

**Authors:** D. Fontes, J. Reyes, K. Ahmed, M. Kinzel

**Affiliations:** 1 Florida Space Institute, University of Central Florida, Orlando, Florida 32826, USA; 2 Mechanical and Aerospace Engineering Department, University of Central Florida, Orlando, Florida 32816, USA

## Abstract

Recent studies have indicated that COVID-19 is an airborne disease, which has driven conservative social distancing and widescale usage of face coverings. Airborne virus transmission occurs through droplets formed during respiratory events (breathing, speaking, coughing, and sneezing) associated with the airflow through a network of nasal and buccal passages. The airflow interacts with saliva/mucus films where droplets are formed and dispersed, creating a route to transmit SARS-CoV-2. Here, we present a series of numerical simulations to investigate droplet dispersion from a sneeze while varying a series of human physiological factors that can be associated with illness, anatomy, stress condition, and sex of an individual. The model measures the transmission risk utilizing an approximated upper respiratory tract geometry for the following variations: (1) the effect of saliva properties and (2) the effect of geometric features within the buccal/nasal passages. These effects relate to natural human physiological responses to illness, stress, and sex of the host as well as features relating to poor dental health. The results find that the resulting exposure levels are highly dependent on the fluid dynamics that can vary depending on several human factors. For example, a sneeze without flow in the nasal passage (consistent with congestion) yields a 300% rise in the droplet content at 1.83 m (≈6 ft) and an increase over 60% on the spray distance 5 s after the sneeze. Alternatively, when the viscosity of the saliva is increased (consistent with the human response to illness), the number of droplets is both fewer and larger, which leads to an estimated 47% reduction in the transmission risk. These findings yield novel insight into variability in the exposure distance and indicate how physiological factors affect transmissibility rates. Such factors may partly relate to how the immune system of a human has evolved to prevent transmission or be an underlying factor driving superspreading events in the COVID-19 pandemic.

## I. INTRODUCTION

By September 2020, the COVID-19 pandemic had infected over 30.6 million people and led to almost one million deaths.^1^ Furthermore, several economic and social implications arose due to the pandemic.^2^

In an attempt to reduce the transmissibility rate, avoiding overload of the health care system, the World Health Organization (WHO), the Centers for Disease Control and Prevention (CDC), and other agencies suggested the practice of physical distancing and the use of face masks. These recommendations rely on studies that did not evaluate the multiphase interaction during respiratory events (speech, coughs, and sneezes). The studies of Wells^3^ and Olsen *et al.*^4^ are frequently used to justify the 1 m–2 m (3 ft–6 ft) physical distancing guidelines. Wells^3^ evaluated the lifetime of suspended droplets falling from 2 m. The study highlights that the spray distance does not exceed a few feet for 2 m falling droplets. Nevertheless, the study did not consider the interaction between the flow from a respiratory event (sneeze/cough) and the surrounding air. The second study investigated the transmission of SARS-CoV-2 on aircraft. The authors found that the majority of people seated 2 m around the infected person became ill, mainly if they were seated in front of or in the same row as the infected person. However, the authors did not provide multiphase analysis of airflow and particle transport.

Recent experimental and numerical studies have provided a better description of pathogen transmission and how it is affected by ambient factors (temperature, humidity, and ambient flows).^5–8^ Dbouk and Drikakis^5^ investigated numerically droplet transport subjected to mass transfer (evaporation) under different environmental conditions. The authors showed that 2 m social distancing may be effective in a quiescent external ambient. However, for wind speed varying from 4 km/h–15 km/h, the droplets may travel up to 6 m. Dbouk and Drikakis^6^ developed theoretical correlations for the unsteady evaporation of coronavirus contaminated saliva droplets. The authors investigated the viability of virus survival through a numerical modeling of cough considering the contaminated saliva droplets. They found that high temperature and low relative humidity reduce virus viability, while high relative humidity can keep significant virus viability at the temperature considered in the study. Prasanna Simha and Mohan Rao^9^ and Verma, Dhanak, and Frankenfield^10^ investigated the effectiveness of mask and hand or elbow obstruction on droplet dispersion from respiratory jets. Verma, Dhanak, and Frankenfield^10^ investigated experimentally the effectiveness of different materials and designs in reducing droplet dispersal. According to the authors, loosely folded face masks and bandana-style coverings provide low blockage for the smallest respiratory droplets, while well-fitted homemade masks with multiple layers of quilting fabric and off-the-shelf cone style masks proved to be the most effective in reducing droplet dispersal. Also, considering the role of masks and obstructions, Prasanna Simha and Mohan Rao^9^ assessed experimentally the maximum spray distance of typical human coughs. Their results showed that the best mask type reduced the spray distance from a cough by more than ten times compared to coughs without mask.

Several parameters affect droplet transport from different respiratory events leading to a high variability of simple spray characteristics, such as spray distance. A recent review compared the predicted horizontal droplet distance of ten relevant experimental, numerical, and analytical studies showing a difference in the horizontal droplet distance for the full spectrum of the droplet size of more than eight times.^11^ The difference in the spray distance is not only affected by external variables, as discussed above. Individual characteristics of the upper respiratory tract (URT) change the internal flow pattern and consequently the droplet formation and airflow velocity at nostrils/mouth exits. Two numerical studies^12,13^ of single-phase airflow during a sneeze showed the changes in the pressure and velocity fields due to nostrils/mouth obstructions and inlet flow variations. Rahiminejad *et al.*^12^ numerically investigated the velocity and pressure fields inside the URT during a sneeze under conditions with open and closed nose or mouth passages. Their results showed that suppressing the nose or mouth increases the pressure inside the URT from 5 to 24 times compared to a sneeze without obstructions. Mortazavy Beni, Hassani, and Khorramymehr^13^ investigated the effect of high and low inlet pressure on the velocity and pressure field in the URT. The authors identified that high inlet pressure may cause some damage to the soft tissue of the URT.

The spray formation from a respiratory event occurs in a relatively short-time period involving complex multi-phase phenomena.^14,15^ A nearly pulsed airflow of high-velocity^16,17^ transports the droplets from the respiratory system to the ambient environment through the nasal and buccal passages. During this event, tissue structures interact with the liquid film adhered to the surfaces of mouth and nose, being sheared by the airflow, ultimately creating a cloud of a large range droplet size. Many numerical and experimental studies have analyzed the resultant spray for different respiratory events and the associated potential of virus transmission.^14,15,18–21^ However, there is a lack of knowledge about the influence of individual characteristics of human physiology on the potential of virus transmission. In this sense, it is crucial to have a deeper understanding of the dependency of these characteristics that may produce variability of the spray formation during respiratory events. In the context of pathogen transmission within populations, the pathogen transmission follows quite well the empirical rule that 20% of the individuals in a given group of people would contribute at least 80% to the transmission potential of the pathogen.^22^ However, in many infectious disease cases, the rate of pathogen transmission was significantly increased by few individuals known as super-spreaders.^23^ The COVID19 pandemic exhibited different transmission rates, which could be related to cascading super-spreading events.^24^ Among several factors that might be related to the pathogen transmission potential of an individual, Hattis *et al.*^25^ and Wong *et al.*^26^ considered the physiological factors relevant in the context of super-spreaders. These physiological factors may influence the geometry of nasal and buccal passages and mucus film properties, which drastically affect the spray formation during a sneeze event.

The geometries of nasal and buccal passages vary depending on the anatomy and pathophysiology characteristics of each person.^27,28^ These features affect the evolution of the flow during a respiratory event. For instance, the air velocity coming out of the mouth during a sneeze increases when the nasal passage is closed (which might be a result of an allergy, hay fever, or septum deviation). In the same way, one considers the obstruction caused by the frontal teeth, which also changes the flow direction. Besides these geometrical changes, the physical properties of the saliva can also modify the overall spray during a sneeze by increasing or reducing the chance of primary or secondary droplet breakup. Fluid properties of saliva have been documented to relate to a variety of human factors.^29–32^ For instance, women tend to have thicker saliva than men (around 50%).^31,33^ Additionally, specific scenarios such as stress and illness tend to drive a human to develop thicker saliva.^27,31^ The changes in the saliva properties affect the multi-phase condition associated with the formation of the primary droplets from the mucus film. Furthermore, the surface tension coefficient and density play an important role in the primary and secondary droplet breakup, since the Weber, $We=\left(\rho_{g}v_{rel}^{2}D\right)/\sigma$, and Ohnesorge, $Oh=\mu_{l}/\sqrt{\rho \sigma D}$, numbers (dimensionless numbers that represent the mechanisms behind droplet breakup) are dependent on these properties.

The present work shows a numerical study of the spray formation of a human model sneezing. Four geometrical conditions of the nasal and buccal passages were considered in an approximated model that includes the throat, nasal, and buccal passages. The spray characteristics of the sneeze spray are evaluated for three saliva types (thinner, base saliva, and thicker), considering droplet distribution affected and not affected by the saliva property changes. The analyses made in this work provide valuable information on how the human physiology factors affect droplet transmission of pathogens.

## II. METHODS

This section includes the models and methods used to analyze the spray generated from a sneeze. First, the geometrical features of the domain, boundary conditions, and hypothesis are described in detail. Considering the physical modeling description, Subsection II A presents the mathematical equations used to represent the gas flow and droplet transport. Finally, the numerical methods and modeling are presented along with the features of the numerical mesh.

### A. Physical modeling

**FIG. 1.**
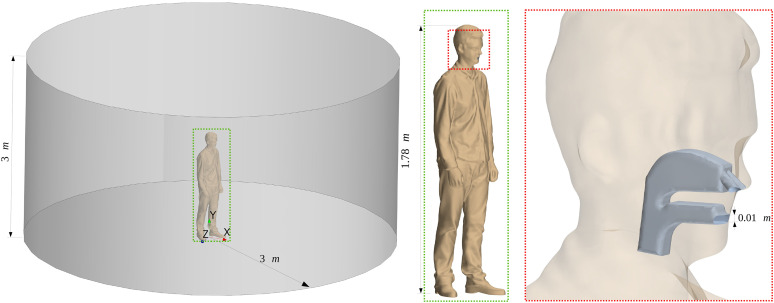
Geometry of the physical domain, highlighting the features and dimensions of the human body and URT.

The physical domain consists of the geometry of a human body centered within a cylindrical room with a radius and a height of 3 m. Figure 1 shows the characteristics of the human body placed in the room, as well as the general features of the simplified upper respiratory tract model. The URT model was built based on general geometrical features of an anatomic URT, including the simplified representation of the pharynx, nasal cavity, and buccal cavity. The dimensions of the simplified model follows typical linear measurements of URT via a computed tomography technique.^34^ The nostrils have a diameter of ∼0.01 m, while the mouth exit is nearly rectangular with a 0.025 m width and a 0.01 m height. Such a geometry provides a reasonable representation to study the effects of various fluid-dynamics aspects to the human sneeze.

**FIG. 2.**
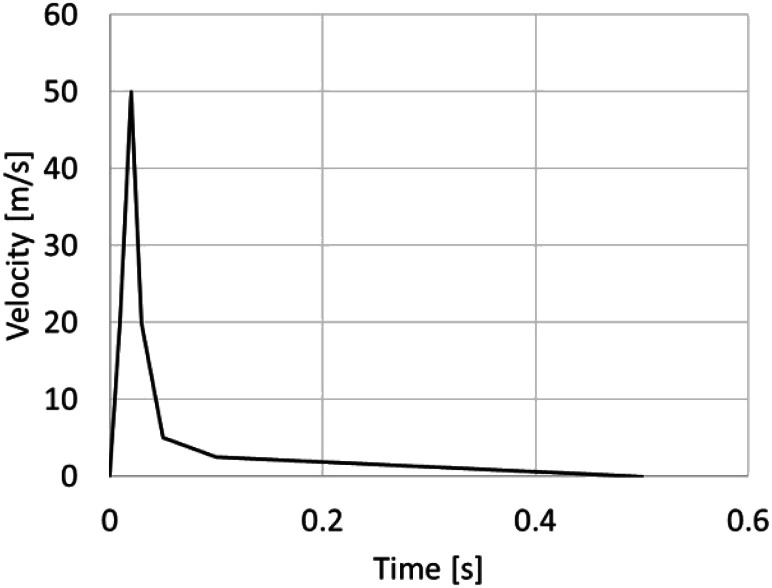
Temporal velocity of the air injected in the bottom surface of the throat.

**FIG. 3.**
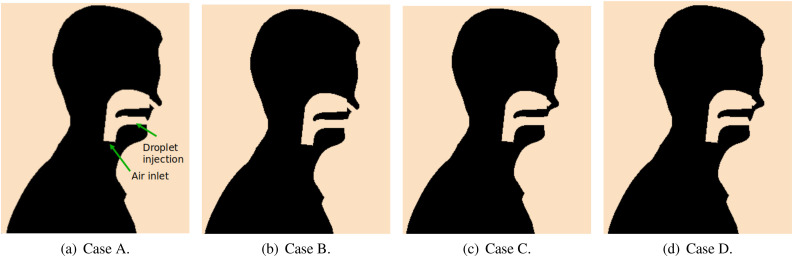
Geometries of URT considering the (a) open nasal passage with teeth, (b) open nasal passage without teeth, (c) blocked nasal passage without teeth, and (d) blocked nasal passage with teeth.

The sneeze event initiates at *t* = 0 s with airflow coming from the bottom surface of the throat within the upper respiratory tract (URT), as indicated in Fig. 3(a). The airflow enters the URT as a planar velocity profile, whose magnitude value is a function of time according to the curve shown in Fig. 2. All the cases evaluated in this work used this same velocity profile, ensuring the same total amount of air ejected as the criterion of comparison. The peak velocity is 50 m/s, and the total duration of the airflow injection is 500 ms. This velocity profile has similar characteristics found in the time function flow dynamics of a cough.^17^ It was corrected to match a typical value of the total volume of air expelled, 0.0012 m^3^ (1.2 l), during a sneeze.^16,19^ The air coming out the nasal and buccal passages has higher momentum and temperature (36.5 °C) than the stationary air in the room, whose temperature is *T*_*amb*_ = 23 °C. This difference in temperature leads to the upward movement of the hotter air due to the buoyancy-weight effect. Both air and droplets coming out the URT are subjected to a gravitational acceleration, $\vec{g}=9.81\text{ m/s}$. Air properties are kept constants, except the density, which is a function of temperature according to the ideal gas equation. The droplets are subjected to secondary breakup, which is the only mechanism leading to droplet size changes. The present work does not consider mass transfers from droplet–droplet (coalescence/stripping) or droplet–environment (evaporation) interactions. The hypothesis of droplets not interacting with each other is valid in a dilute concentration region. However, it may present deficiencies inside the URT and close to the mouth/nasal passages, which are neglected in the present work. The hypothesis of no evaporation is consistent with an external environmental condition with a high relative humidity along with a low ambient temperature (*T*_*amb*_ = 23 °C), which does not allow significant droplet mass loss due to evaporation. In a recent investigation about the weather impact on airborne coronavirus survival,^6^ the authors found that the spray distance and droplet mass loss is less affected in ambient with a low temperature (considering a temperature range of 0 °C–40 °C) and a high relative humidity.

Considering that the droplets are mostly generated from the mucus film inside the mouth, the droplets injection occurs on a surface with 5.96 cm^2^ of area located 3 mm above the bottom surface of the buccal cavity, as depicted in Fig. 3(a), at a constant flow rate that results in a total volume injection of 2.0 ml during the sneeze duration, which corresponds to 0.167% of the airflow injected. This total volume injection was the same for all the cases evaluated in this work.

The physical model considers no-slip conditions at the top, bottom, and human model surfaces. The temperature of the human body surface covered by clothes (*y* < 1.46 m) is 23.0 °C and 36.5 °C for uncovered surfaces (neck and head of the human model). The lateral outer surface is an outlet condition.

In the following, we present the physical modeling associated with nasal/buccal passages and saliva properties.

#### 1. Nasal/buccal passages

The upper respiratory tract (URT) model is a connected flow network between throat, nasal, and buccal passages. Since nasal and buccal passages may present some modifications depending on the natural response of the human body to diseases or an acquired condition,^28,35^ the URT model used in the simulations presents some adaptations to represent some of those modifications. Thus, to investigate different features of nasal and buccal passages on the formation of droplets plume generated during a sneeze, four combinations (Table I) regarding the nasal and buccal passages are used to represent physiological features (Fig. 3). The obstructions caused by teeth are represented by adding a barrier beneath the upper surface of the mouth exit with ≈9 mm in height. Similarly, the nasal passages received extra volumes in the nostrils ducts to work as barriers to the flow.

**TABLE I.** URT geometries considered in the present work and their potential correlations to human physiological factors.

Case name	Geometry	Human physiological factor
Case A	Open nasal passage with teeth	Baseline
Case B	Open nasal passage without teeth	Poor dental health
Case C	Blocked nasal passage without teeth	Poor dental health/congested
Case D	Blocked nasal passage with teeth	Congested

#### 2. Saliva properties

In order to evaluate the effect of saliva, a sensitivity study that varies the fluid density, viscosity, and surface tension coefficient is performed. Three cases are considered: (1) saliva (based on water properties), (2) thicker saliva, and (3) thinner saliva. The fluid properties of saliva are increased/reduced by factors of 20%, 30%, and 50% for the density, dynamic viscosity, and surface tension coefficient, respectively. The overall values evaluated are depicted in Table II.

**TABLE II.** Physical properties of the fluids considered in the present study.

Property	Air	Thinner saliva	Saliva	Thicker saliva
*ρ* (kg/m^3^)	*P*_*amb*_/(*RT*)	798.05	997.56	1197.07
*μ* (Pa s)	1.86 × 10^−5^	6.20 × 10^−4^	8.90 × 10^−4^	11.50 × 10^−4^
*σ* (N/m)	…	0.036	0.072	0.108
Human physiological				
factor	…	Male not stressed	Female not stressed	Person under stress

**FIG. 4.**
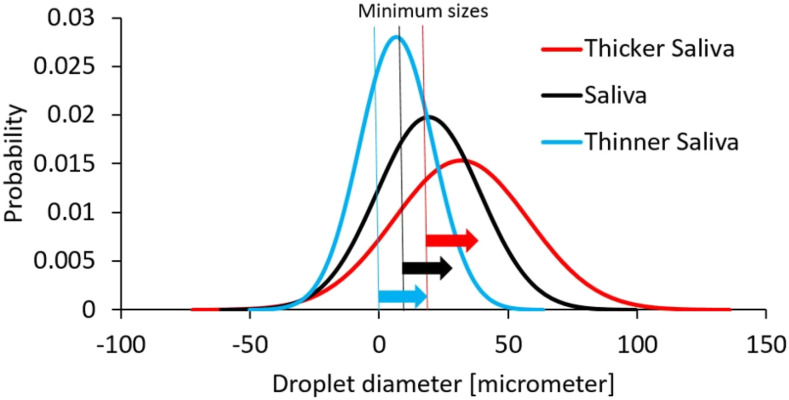
Droplet distributions for different saliva properties.

The present studies are designed to develop an understanding of how fluid properties of saliva affect droplet dispersion from a human sneeze. Assuming that the airflow does not change, the fluid properties of saliva relate to the spray dispersion through two droplet breakup mechanisms. The first is the primary breakup associated with the initial formation of droplets within the mouth. This primary breakup mechanism is due to the film-flow instabilities caused by the interfacial interaction between airflow and the mucus film. This mechanism drives the initial droplet size distribution as well as the overall content entrained into the airflow. Sprays can then be driven by secondary breakup mechanisms that occur after the droplets are suspended in the airflow. Such a process is expected to primarily occur within the mouth and just external to the mouth. This secondary breakup is driven by flow instabilities developed from aerodynamic shear and pressure forces that lead to droplet break up and atomization. In this effort, the studies are devised to understand sensitivities associated with primary and secondary breakup mechanisms. In order to evaluate primary breakup mechanisms, we consider three distinguished droplet distributions for the three salivary fluids, where the primary breakup affects the initial droplet size distribution. This effect is depicted in Fig. 4. These distributions are based on some experimental measurements of the droplet size generated in sneeze tests considering saliva property modifications. In order to focus the study on the secondary breakup, the input droplet size distribution is fixed (to the saliva distribution) and the secondary droplet processes are modeled. The sensitivity to each of these studies will yield insight into the importance of each breakup process.

### B. Mathematical modeling

The model equations used to represent the physical scenario can be described as an Eulerian gas flow one-way coupled to Lagrangian saliva droplets. Accordingly, the gas phase is solved as a continuous phase through mass, momentum, and energy balance equations, represented by the following partial differential equations in index notation:

$$\frac{\partial \rho }{\partial t}+\frac{\partial \rho {û}_{i}}{\partial x_{i}}=0,$$ (1)

$$\frac{\partial \rho {û}_{i}}{\partial t}+\frac{\partial \left(\rho {û}_{i}{û}_{j}\right)}{\partial x_{j}}=-\frac{\partial \hat{p}}{\partial x_{i}}+\rho \vec{g}+\frac{\partial \left(\mu {Ŝ}_{ij}+Tm_{ij}\right)}{\partial x_{j}},$$ (2)

$$\frac{\partial \rho c_{p}\hat{T}}{\partial t}+\frac{\partial \left(\rho c_{p}{û}_{i}\hat{T}\right)}{\partial x_{j}}=k\frac{\partial^{2}T}{\partial x_{j}}.$$ (3)

In Eqs. (1)–(3), *ρ* is the density, which is a function of the temperature according to the ideal gas equation, ${û}_{i}$ is a filtered velocity field, $\hat{p}$ is the pressure field, ${Ŝ}_{ij}=\left(\frac{\partial u_{i}}{\partial x_{j}}+\frac{\partial u_{j}}{\partial x_{i}}\right)$ is the shear stress tensor, $Tm_{ij}=f_{\Delta }\left(\frac{\Delta }{l_{k}}\right)2\mu_{t}{Ŝ}_{ij}$ is the Reynolds stress tensor, which is a function of a damping function from the Detached Eddy Simulation (DES) model, the local measure of the grid size, Δ, the turbulent length-scale, *l*_*k*_, the turbulence viscosity, *μ*_*t*_, and the shear stress tensor, $\hat{T}$ is the filtered temperature, and *c*_*p*_ and *k* are the specific heat and thermal conductivity coefficients of the air, respectively.

In the momentum equation [Eq. (2)], all the variables are filtered based on the Detached Eddy Simulation (DES) model.^36^ The DES model is a hybrid turbulence model that uses unsteady Reynolds averaged Navier Stokes (URANS) and the large-eddy simulations (LES) equations. DES uses URANS in boundary layers and irrotational flow regions. For regions with mesh sufficiently refined, the turbulence model is modified to emulate a basic LES subgrid-scale model in detached flow regions. The elliptic *k* − ε turbulence closure model is to used to solve the Reynolds stress tensor, $T_{m_{ij}}$, in the DES model.

The droplets are solved via a Lagrangian approach. The model is driven by Newton’s second law to calculate the droplet acceleration coupled to aerodynamic drag ($F_{d_{i}}$), lift ($F_{l_{i}}$), buoyancy/weight ($F_{{w,b}_{i}}$), and pressure gradient forces ($F_{p_{i}}$). Droplet velocity and position are obtained from the solution to the following ordinary differential equations:

$$m_{d}\frac{du_{d_{i}}}{dt}=F_{d_{i}}+F_{l_{i}}+F_{{w,b}_{i}}+F_{p_{i}},$$ (4)

$$\frac{dx_{d_{i}}}{dt}=u_{d_{i}},$$ (5)

where the subscript *d* refers to droplet, *u* and *x* are the droplet velocity and position, respectively, *m* is the droplet mass, and subscript *i* indicates the three components of a vector. Other forces such as virtual mass and Basset forces are normally not relevant because of the high liquid/air density ratio.

The drag force is calculated according to the following equation:

$$F_{d_{i}}=m_{d}\frac{3\rho C_{D}}{4\rho_{d}d_{d}}\left|u_{i,t}-u_{d_{i}}\right|\left(u_{i,t}-u_{d_{i}}\right).$$ (6)

The drag coefficient, *C*_*D*_, is based on the correlation of Schiller and Naumann,^37^ which yields

$$C_{D}=\left\{\begin{array}{cc} \frac{24}{Re_{d}}\left(1+\frac{1}{6}Re_{d}^{2/3}\right),\; & Re_{d}\leq 1000,\\ 0.424,\; & Re_{d}>1000, \end{array}\right.$$ (7)

where $Re_{d}=\frac{\rho |u_{i}-u_{d_{i}}|d_{d}}{\mu }$ is the droplet Reynolds number. The shear lift force^38^ is a perpendicular force to the relative motion of the droplet and the flow. It arises when there is a velocity gradient in the fluid orthogonal to the relative motion of the droplet,

$$F_{l_{i}}=C_{L}\frac{\rho \pi }{8}d^{3}\left(\vec{v_{r}}\times \vec{\omega }\right),$$ (8)

where $\vec{v_{r}}$ is the relative velocity between the droplet and the gas flow, $\vec{\omega }=\nabla \times \vec{v}$ is the curl of the gas flow velocity, and *C*_*L*_ is the shear lift coefficient, which is calculated through the Sommerfeld^39^ equation,

$$C_{L}=\frac{4.1126}{Re_{S}^{0.5}}f.$$ (9)

The lift shear coefficient is dependent on the Reynolds number of the droplet, *Re*_*d*_, and the Reynolds number of the of the shear flow, $Re_{S}=\frac{\rho d^{2}|\omega |}{\mu }$, through the function, *f*,

$$f=\left\{\begin{array}{cc} \left(1-0.3314\beta^{0.5}\right)e^{-0.1Re_{d}}\;\\ +0.3314\beta^{0.5},\; & Re_{d}\leq 40,\\ 0.0524\left(\beta Re_{d}\right),\; & Re_{d}>40, \end{array}\right.$$ (10)

where $\beta =\left(0.5\frac{Re_{S}}{Re_{d}}\right)$. The combined buoyancy-weight force is calculated according to the following equation:

$$F_{{w,b}_{i}}=\left(1-\frac{\rho }{\rho_{d}}\right)m_{d}g_{i}.$$ (11)

The last term of forces considered in the droplet motion equation is the pressure gradient term force, which is related to the gradient of pressure of the gas flow acting on the volume of the droplet, *V*_*d*_,

$$F_{p_{i}}=-V_{d}\nabla p.$$ (12)

### C. Numerical modeling

The gas flow is solved numerically in an unstructured mesh using the finite volume method^40^ to discretize the balance equations. A second-order scheme is used for the transient, while a bounded central-differencing scheme^41^ was used for the spatial interpolations. The SIMPLE method^40^ along with the momentum interpolation method of Rhie and Chow^42^ numerically couples the pressure and velocity fields for the variables that are stored in a collocated grid arrangement. The temperature of the air flow is solved through a segregated solver, considering a second-order convection and secondary gradients.

**FIG. 5.**
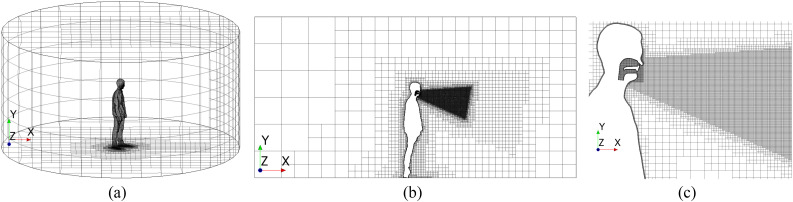
Numerical mesh used to discretize the physical domain, highlighting the regions with higher refinement: (a) full domain, (b) lateral view, and (c) zoomed-in lateral view.

The mesh used to discretize the domain is composed of trimmed hexahedral cells and prism layers at the surfaces of the human body and URT (no-slip condition). Figure 5 shows the general view of the mesh used in the present work for all cases. Prism layers in the vicinity of the human body and URT surfaces are intended to capture the boundary layer development. To accurately capture different *y*^+^ conditions, a wall treatment with a blended function is used. The final mesh is refined in a conical region where the turbulent structures might develop and in the region inside the URT. The final mesh is composed of 2.4 × 10^6^ cells.

Additionally, an adaptive time step was used considering a time step range of Δ*t* = 0.0001 s–0.01 s, where Δ*t* adapted to the lesser of a maximum convective Courant number ($Co=\left|u_{i}\right|\frac{\Delta t}{\Delta x}$) of 20.0 and the mean of 0.75.

The droplets are solved considering a statistical approach based on the parcel concept, where each parcel represents a number of real droplets with the same state (position and velocity) and diameter. This approach reduces the number of actual particles being solved numerically, which lowers the computational cost associated with the numerical solution of spray formed from the sneeze cases. The position and velocity of the droplets are calculated using a tracking integration method of first order, which was enough to track the droplet plume, since the droplets are not subjected to abrupt changes in the flow field.

The numerical modeling for both gas and droplet phases are implemented in the context of the commercial software Star-CCM+,^43^ which was used in the present work.

## III. RESULTS

Results are focused on two distinct physiological studies. The first is oriented on understanding the effects of the buccal and nasal passage geometries. The second focuses on effects associated with the fluid properties of saliva. The studies interrogate both the flow and particle characteristics in detail by comparing factors relating to potential transmission of airborne pathogens.

### A. Effect of the buccal and nasal passages

This section presents a sensitivity study of the jet evolution and particle dispersion from a sneeze event for the four different nasal and buccal passages geometries indicated in Fig. 3. This sensitivity study presents important information regarding geometrical differences of the nasal and buccal passages that might be due to disease, health, or differences between individuals. All these cases use the same baseline, saliva character.

**FIG. 6.**
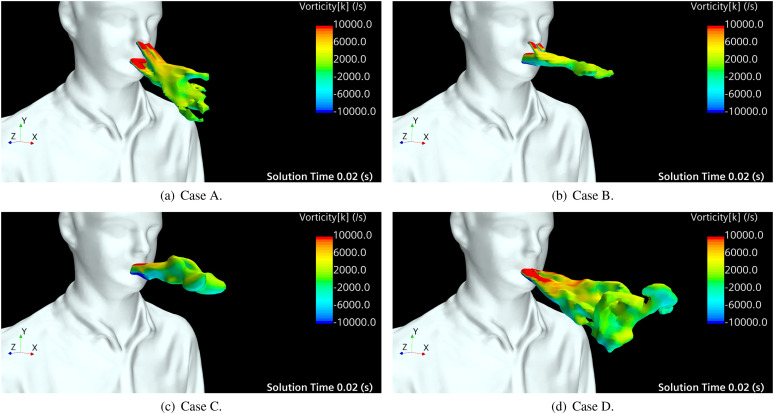
Iso-surfaces of velocity magnitude, $|\vec{v}|=40\text{ m/s}$, when the sneeze velocity is maximum (*t* = 0.02 s), colored by vorticity at the *z*-axis: (a) open nasal passage with teeth, (b) open nasal passage without teeth, (c) blocked nasal passage without teeth, and (d) blocked nasal passage with teeth.

Figure 6 shows the iso-surfaces of a velocity magnitude of $|\vec{v}|=40.0\text{ m/s}$ colored by the vorticity in the *z*-axis at *t* = 0.02 s after the beginning of the sneeze that corresponds to the time in which the injected air velocity is maximum (Fig. 2). Considering the topology of jets at the initial stage of the sneeze for the four cases, we identified the mechanism of jet redirection. Jet redirection occurs due to the combined effect of teeth and nasal jets. Comparing cases A–D, the obstruction caused by the upper teeth is more influential on redirecting the main flow from the mouth than the nasal jets. In B, the nostrils flow slightly modifies the main flow trajectory, but it does not have enough momentum to produce similar changes in the flow trajectory as those caused by teeth. A secondary mechanism that plays a role in the jet topology is related to the aerodynamic instabilities and recirculation of the surrounding air, induced by the jet flows. The z-vorticity field shown in Fig. 6 indicates that the jets from the mouth and nostrils promote swirling flows of air in a counterclockwise direction in the upper side and a clockwise direction in the bottom side of the main flow. This recirculation would contribute to transversely spread and slowdown of the flow jet. However, to better understand the influence of the vortex structures and the aerodynamic instabilities on lateral spreading of the main jet, a large Eddy simulation analysis of these cases should be performed.

**FIG. 7.**
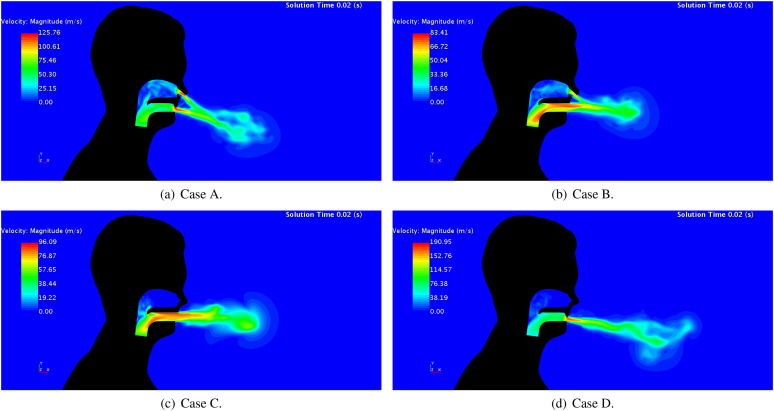
Lateral views of velocity magnitude when the sneeze velocity is maximum (*t* = 0.02 s): (a) open nasal passage with teeth, (b) open nasal passage without teeth, (c) blocked nasal passage without teeth, and (d) blocked nasal passage with teeth.

Figure 7 shows the velocity magnitude in a longitudinal plane inside the URT and the surrounding region of the human head for the four cases when the sneeze velocity is maximum (*t* = 0.02 s). This figure highlights that either the blockages on nasal passages or the reductions of the buccal passage area cause an increase in the flow velocity. Obstruction of the nasal passage increases the maximum flow velocity in 52% and 15%, considering a mouth with and without teeth, respectively. On the other hand, a mouth with teeth increases the maximum flow velocity by approximately half and double for open and blocked nasal cavities, respectively.

**FIG. 8.**
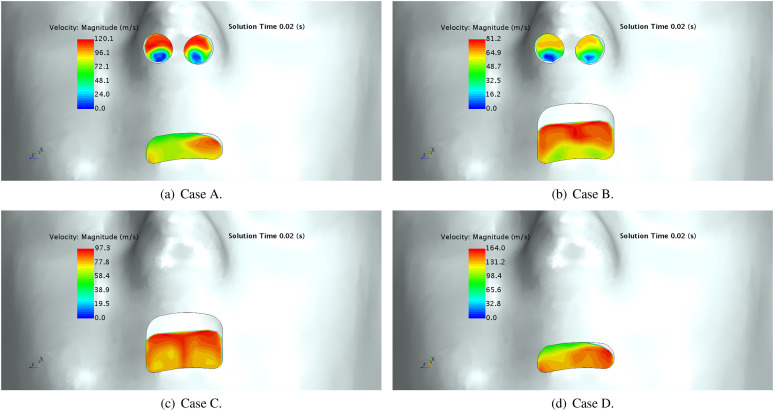
Frontal views of the velocity magnitude when the sneeze velocity is maximum (*t* = 0.02 s): (a) open nasal passage with teeth, (b) open nasal passage without teeth, (c) blocked nasal passage without teeth, and (d) blocked nasal passage with teeth.

As depicted in Fig. 8, the airflow at the exit sections of nasal and buccal cavities indicates important differences that have potential to change droplets extracted from the mucus film. In assessing the detailed velocity distribution in Fig. 8, it can be observed that there is an increased velocity in the lower regions of the mouth when there is a restricted exit area due to teeth (see cases A and D). Although the present work did not model the primary breakup of the mucus film, these flow velocity characters are anticipated to drive film stripping. Assuming that gravity drives a thicker mucus film on lower buccal surfaces, there is a potential for an increased mucus combined with high velocities that can drive increased amounts of droplets. Alternatively, Cases B and C present their highest velocities on upper regions of the exit plane, where it is expected that mucus films are thinner. Considering results of these configurations, buccal cavities without teeth may lead to fewer droplets than those with teeth.

**FIG. 9.**
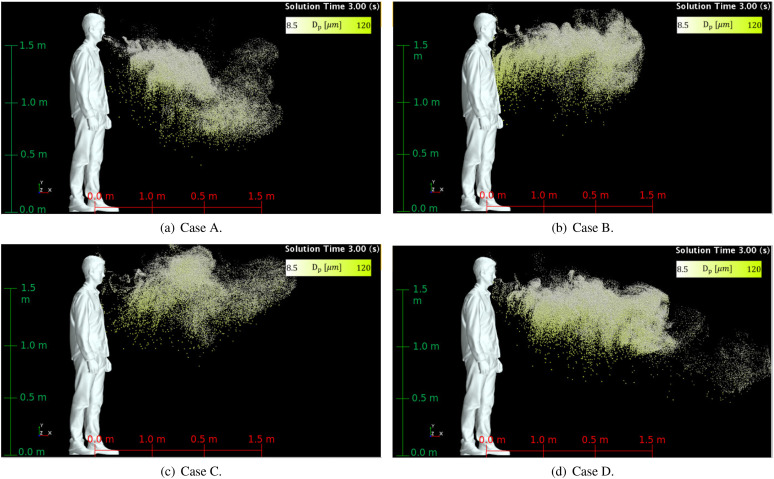
Droplets dispersion for different nasal/buccal passages at *t* = 3.0 s: (a) open nasal passage with teeth, (b) open nasal passage without teeth, (c) blocked nasal passage without teeth, and (d) blocked nasal passage with teeth.

The potential for pathogen transmission can be assessed through the qualitative and quantitative information of the generated spray from a sneeze for the different URT geometries. Figure 9 shows the droplets dispersion at *t* = 3 s for the four buccal/nasal passages considered in this work. The droplet diameter is represented by a linear scale of color and size for the droplets. This figure qualitatively presents information on the generated spray from a sneeze for the four URT geometries considered in this work.

**FIG. 10.**
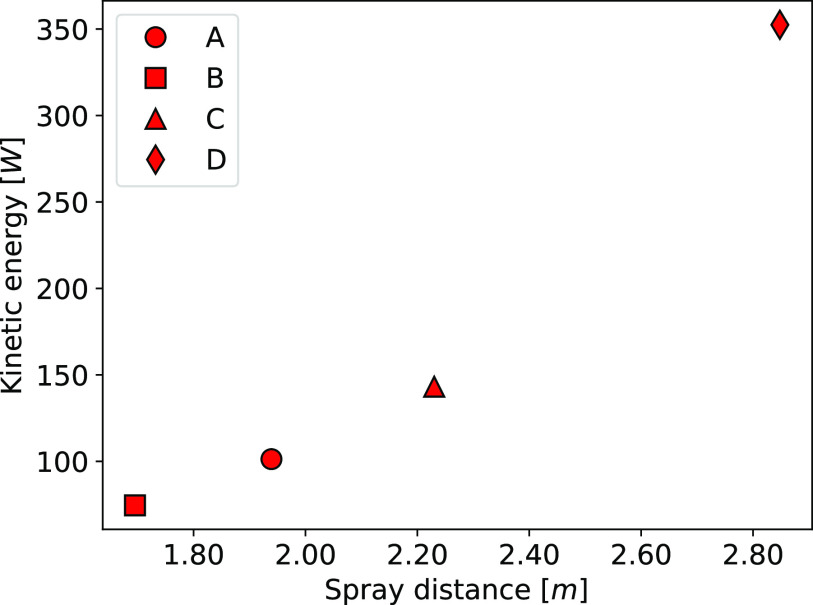
Relation between the peak of kinetic energy and the horizontal distance of the spray edge 5 s after the sneeze.

The distance the droplets travel is directly dependent on the kinetic energy of the airflow delivered at the mouth exit. To highlight this dependency, Fig. 10 shows the relation between the horizontal distance of the spray edge at 5 s after the sneeze and the mean kinetic energy in the mouth exit when the sneeze velocity is maximum (*t* = 0.02 s). The kinetic energy required to increase the spray distance grows nearly following a quadratic function. This relation highlights how influential the biological differences are, since they may produce a variation in the average value of the kinetic energy peak at the mouth surface over 250 W. The resultant direction of the sneeze flow is related to the flow redirection caused by teeth or nasal jets. Based on the horizontal distance (red axis), the droplets go furthest according to the following order D, C, A, and B. In terms of height reached by the droplets, the cases are ranked as follows: C, B, A, and D. Due to the upper teeth restriction (cases A and D), the droplets are transported toward the ground by the airflow from the sneeze, which leads to a higher number of droplets falling below the 1.0 m height at 3 s after the sneeze compared to the cases B and C.

In Fig. 9, one can see droplets above the height of the human model, which is due to the thermal buoyancy mechanism carrying small droplets close to the human face. The thermal plume formed due to the higher temperature of the head and neck promotes a weak ascending movement of the smaller droplets that remained close to the human model face. An external flow may transport these smaller droplets to longer distances or even reach air conditioner ducts. The higher temperature of the air exhaled from the lungs does not promote a significant upward transport of the droplets since it rapidly exchanges energy with the room ambient, and the momentum of the airflow is a more dominant mechanism to advect the droplets.

**FIG. 11.**
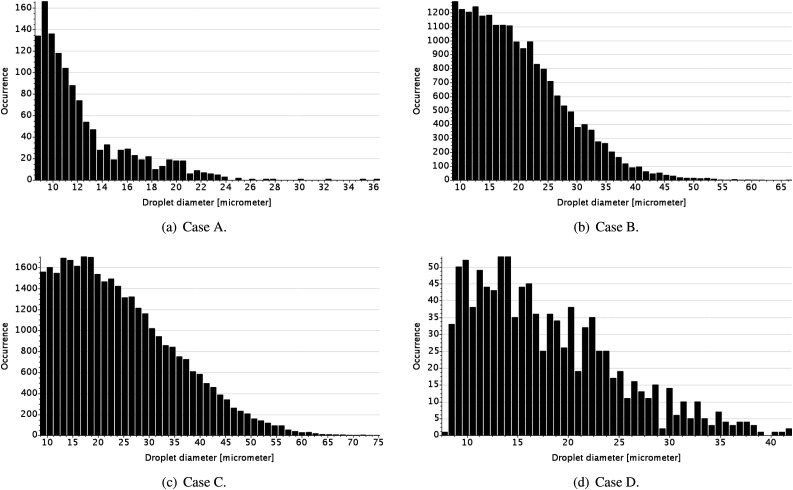
Histograms of droplet occurrence in a height greater than 1.6 m for different nasal/buccal passages at *t* = 3.0 s: (a) open nasal passage with teeth, (b) open nasal passage without teeth, (c) blocked nasal passage without teeth, and (d) blocked nasal passage with teeth.

**FIG. 12.**
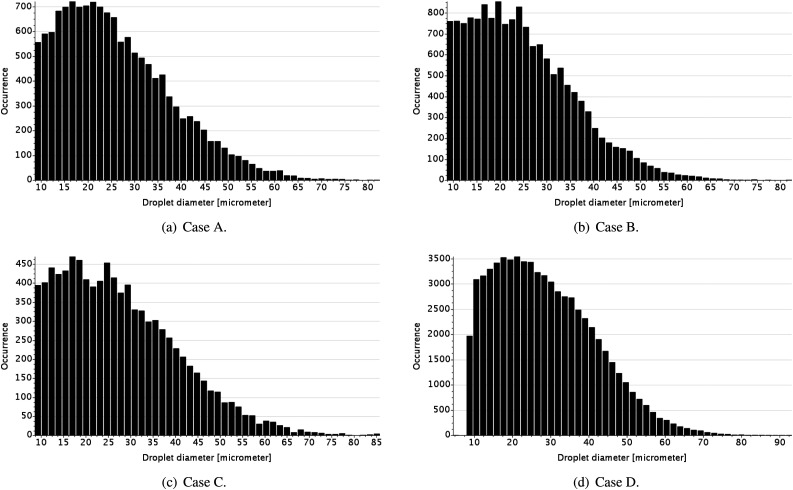
Histograms of droplet occurrence in a distance greater than 1.22 m (4 ft) for different nasal/buccal passages at *t* = 3.0 s: (a) open nasal passage with teeth, (b) open nasal passage without teeth, (c) blocked nasal passage without teeth, and (d) blocked nasal passage with teeth.

To quantify the potential for airborne pathogen transmission for the four different cases, we examined the histograms of droplet occurrence for heights greater than 1.6 m (Fig. 11) and for a horizontal distance greater than 1.22 m (4 ft; Fig. 12).

At the height of interest, there is a prevalence of smaller droplets for the four cases, as depicted in Fig. 11. In the cases where there is a flow redirection, caused by either nasal flow or upper teeth, the occurrence of droplets is lower in this height range compared to the cases in which there is no redirection. A comparison of the pair cases B–C shows the effect of nasal flow redirecting the main flow from the mouth. Comparisons of the pair cases A and B and C and D show the redirecting flow effect of the main flow from the mouth due to the teeth. The pair cases A and D comparison demonstrate that the flow carries more droplets toward the ground in D than in A. This occurs because the higher exit velocity in the mouth found in D than that in A has a more intense effect on redirecting the flow compared to the nasal flow effect. The redirection of the main flow by the nasal jets is secondary compared to the teeth effect because the nasal flow has less momentum than that from the mouth, and the interaction between these two flows occurs mostly laterally, as shown in Fig. 6. The total occurrences of droplets for the full range of the droplet diameter for a height higher than 1.6 m at *t* = 3 s for cases A–D are 1245, 20 200, 33 509, and 1058. Considering the droplet with a diameter lower than 10 *µ*m, which is nearly a common threshold found to define an airborne transmission,^44,45^ the order from the lowest to the highest parcel occurrence remains the same as the full range. Case C shows the highest occurrence of droplet parcels (1804), which is ≈15 times higher than the occurrence for case D (124; the lowest one).

**FIG. 13.**
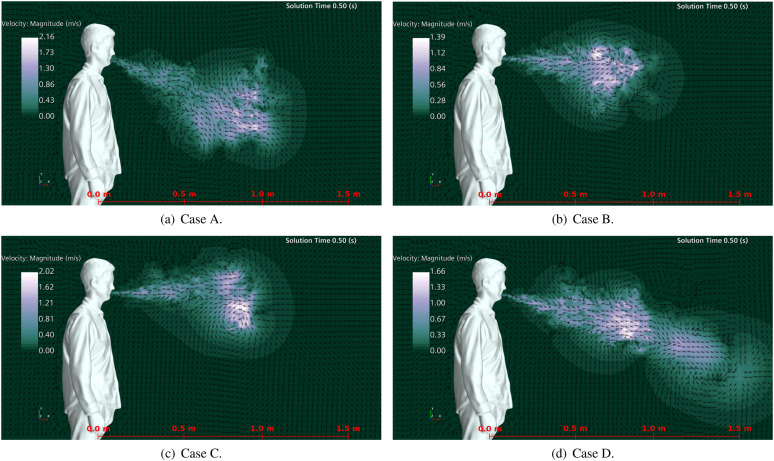
Velocity vector at the end of the sneeze event for different nasal/buccal passages: (a) open nasal passage with teeth, (b) open nasal passage without teeth, (c) blocked nasal passage without teeth, and (d) blocked nasal passage with teeth.

Another important measurement of the pathogen transmission potential from a sneeze event is the droplet distribution at specific distances from the host. Figure 12 presents histograms of the droplet occurrence in a distance greater than 1.22 m (4 ft) at *t* = 3 s. When considering the longitudinal distance, rather than height, the droplet distributions follow a different trend. For instance, case C presents the lowest droplet occurrence compared to the other cases, despite showing the highest total droplet occurrence considering the occurrence analysis for a height greater than 1.6 m. In cases A and B, the airflow from the nasal passages interact with the main flow exhausted from the mouth in two ways: (1) a weak redirection of the flow toward the ground and (2) enhanced mixing of the flow streams. The effect of redirection was analyzed in the droplet occurrence histograms associated with height. The enhanced mixing appears to alter the vortex formation created when the main flow from the mouth enters a stationary flow. In the cases without nasal flow (C and D), the airflow coming from the mouth generates a vortex that initially drives droplets perpendicularly to the main flow. The position of the vortex heading the main flow depends on the velocity and exit diameter of the jet.^46,47^ Thus, depending on the airflow characteristics exiting the mouth, the longitudinal distance the vortex travels may vary and drastically affects the transport of pathogen-carrying droplets. Comparing the vector fields at the end of the sneeze event (*t* = 0.5 s; see Fig. 13) provides a better explanation of the outcome droplet occurrence for each case. The sneeze vector field shows that some cases present vortex structures with a velocity magnitude either higher or lower than that of the bulk flow. Vortex structures with a high-velocity magnitude can spread droplets perpendicularly to the main flow. In contrast, those with a low-velocity magnitude have a less important role in spreading droplets during the sneeze. For instance, case C (lowest number of droplets for a distance greater than 1.22 m) has vortex structures with the highest velocities. This condition leads the droplets to spread vertically rather than to move longitudinally. On the other hand, in case D (highest number of droplets for a distance greater than 1.22 m), the vortex structures that would spread droplets perpendicularly to the flow have the smallest values of the velocity magnitude.

### B. Effect of the liquid droplet properties

The study explores the potential for pathogen transmission due to the fluid properties of saliva through the characterization of droplets. The baseline URT geometry is fixed (case A) for the analysis of the three saliva types (thinner saliva, saliva, and thicker saliva) described previously in Sec. II A.

To estimate the effect of the saliva properties on the primary breakup of the liquid film, we compare the spray formed from the sneeze using the same droplet distribution for the three saliva types and three different droplet distributions. The distributions used for the saliva and the thicker saliva were estimated based on the experimental measurements of sneezes when the properties of the saliva of a person were modified by adding some ingredients to the mucus within the mouth. The droplet distribution used for the thinner saliva is an extrapolation from the droplet distributions of saliva and thicker saliva.

**FIG. 14.**
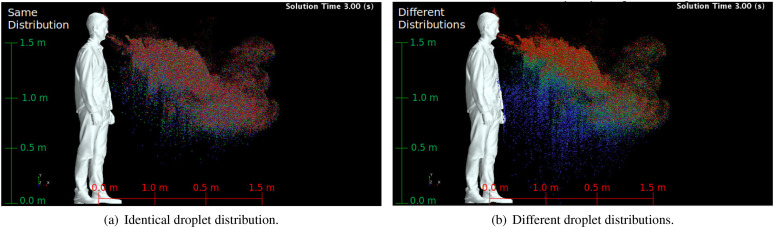
Droplets dispersion considering (a) a unique droplet size distribution and (b) three different size distributions at the injection for three different saliva properties at *t* = 3.0 s. Droplets are colored according to the saliva properties: blue represents thicker saliva, green represents saliva, and red represents thinner saliva.

Figure 14 shows the lateral view of droplets dispersion for saliva, thinner saliva, and thicker saliva using identical and different droplet distributions at the injection at 3 s after the sneeze. In this figure, thicker saliva, saliva, and thinner saliva are represented by blue, green, and red, respectively. When fixing the initial droplet distribution [Fig. 14(a)], the spray formed by each saliva type is a result of the differences related to droplet weight and secondary breakup. Regarding the droplet weight, the droplets whose density is higher fall faster and present distinct spatial distributions of the spray as the drag forces are not prevalent over the weight forces. In the cases evaluated, the drag force attenuates its effect on the droplet as soon as the sneeze flow stops, *t* = 0.5 s. For instance, when the airflow from the sneeze is negligible, the heaviest droplets (blue) are closer to the ground compared to the lightest droplets (red) purely due to the differences between densities, as seen 3 s after the sneeze. The secondary breakup affects the character of the generated spray and, consequently, the virus transmission. Depending on the ratio between the inertial forces (aerodynamics) and the cohesion forces (surface tension), the larger droplets may break into smaller droplets generating a higher number of droplets of the spray formed by the sneeze. As the surface tension coefficient is higher for the thicker saliva, one expects that the droplets are less prone to rupture into smaller droplets compared to the thinner saliva, which has the lowest surface tension coefficient. At the final simulation time, *t* = 5 s, taking the saliva as the base, the number of parcels for the thinner saliva and thicker increases and reduces by ≈13% and ≈7.5%, respectively.

The different droplet distribution at the injection [Fig. 14(b)] is a result that represents the formation of the first droplets from the liquid film, besides the weight effect. The different droplet distributions artificially represent the primary breakup assuming that under the same aerodynamics forces, saliva with a higher surface tension coefficient and viscosity values would generated droplets in a range of larger sizes compared to saliva with a lower surface tension coefficient and viscosity values. In Fig. 14(b), the distinction between the generated sprays from different saliva types is more noticeable than that considering the same droplet distribution at the injection. The thinner saliva distribution has smaller droplets, which promote a spray that is suspended for longer time in the air compared to saliva and thicker saliva. Thus, at *t* = 3 s, while the droplets of thicker saliva (blue) are reaching the ground, the droplets of thinner saliva (red) are still being transported by the plumes generated from the sneeze and thermal buoyancy, as seen close to the man’s head. The effect of saliva properties on the virus transmission potential is first dependent on the primary breakup since it provides the droplet distribution leaving the URT. Based on the spray results, the secondary breakup and droplet settling are secondary mechanisms related to the saliva properties on changing the virus transmission potential in a sneeze.

**FIG. 15.**
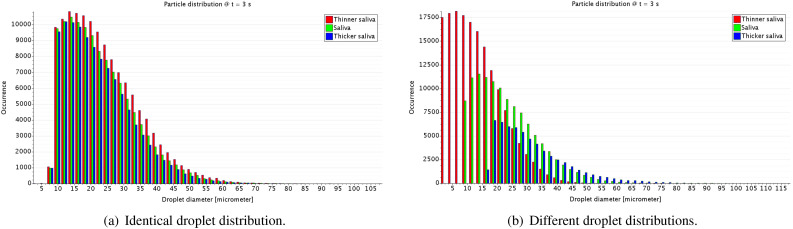
Histograms of droplet occurrence in a height range of (1.0–1.5) m considering (a) an unique droplet size distribution and (b) three different size distribution at the injection for three different saliva properties at *t* = 3.0 s.

The histograms of droplet occurrence in a height range (1.0–1.5) m provides more quantitative information to investigate the virus potential related to different saliva properties. For the identical droplet distribution at the injection, the differences in the droplet occurrence is a result of secondary breakup and settling mechanisms. However, these differences are less important than those related to the primary breakup, as shown in Fig. 15. When the primary breakup is added to the numerical modeling through different droplet distribution at the injection, the droplet occurrence is significantly changed in the height range of (1.0–1.5) m. This height range is critical in terms of virus transmission because it is a range where the majority of the droplets from the sneeze is initially projected and may be either breathed by other person or be transported by an air crossflow. In both situations, the amount of droplets in this height range is highly determinant for direct contamination from person to person, as well as distant virus transmission due to the droplets being transported by a secondary air current with lower momentum than the sneeze.

## IV. CONCLUSIONS

The present work presents numerical analysis of the effect of human physiology factors on droplet transmission of pathogens during a sneeze event. Numerical simulations using a Euler–Lagrange approach are used to represent the airflow and droplet transport for a human body model considering a simplified upper respiratory tract. The numerical simulations consider two physiological factors: (i) geometrical features of nasal/buccal passages and (ii) saliva properties.

The main findings of this work are described as follows:

• Differences in the nasal/buccal passages have a dramatic impact on the spray characteristics that are associated with pathogen transmission rates. For instance, considering that spray distance is an important metric for the virus transmission potential, the obstruction of nostrils increases this metric over 60%.
• The physical properties of the saliva change the occurrence and general aspects of the generated spray after the sneeze event. Considering different droplet distributions at the injection, the droplet occurrence and qualitative characteristics of the spray are substantially changed. The changes are associated with the primary breakup, represented by different droplet distributions, and the secondary breakup mechanism. When a single droplet distribution is considered for both salivary fluids, the differences on the droplet occurrence present a slight difference between fluids. Thus, subtracting the effects of secondary breakup from the simulations considering different droplet distributions, the primary breakup has a major importance on the spray formation than the secondary breakup.

The present work provides useful information on the potential of pathogen transmission. For instance, the results indicate that the body naturally responds to mitigate airborne transmission when stressed or ill. Additionally, women appear to be less likely to transmit airborne pathogens. Finally, a congested/ill host may be less likely to transmit a pathogen when they frequently blow their nose. It is also possible that several human psychological factors favorable to aerosol formation amassed are key to driving the super-spreading events that have been observed in several cases in the COVID-19 pandemic. The present work shows insight into these relationships, but needs to be further investigated for verification.

## Data Availability

The data that support the findings of this study are available within the article.
